# SeSA-HCPT: A dual-targeting agent that induces DNA damage and inhibits repair for castration-resistant prostate cancer therapy

**DOI:** 10.1016/j.isci.2026.114824

**Published:** 2026-01-29

**Authors:** Yajie Wang, Qiuyu Wang, Li Meng, Xiaoying Lian, Xinyue Wu, Yuqing Wang, Tianyu Zhang, ShiLin Wei, Yanming Wang, Changjun Zhu

**Affiliations:** 1 Tianjin Key Laboratory of Animal and Plant Resistance, College of Life Sciences, Tianjin Normal University, Tianjin 300387, China; 2 College of Pharmacy, Key Laboratory of Bioactive Materials for the Ministry of Education, Nankai University, Tianjin 300350, China

**Keywords:** Molecular biology, Biotechnology, Cancer

## Abstract

Castration-resistant prostate cancer (CRPC) remains difficult to treat due to tumor heterogeneity and resistance. We developed SeSA-HCPT, a dual-targeting compound that links the topoisomerase I inhibitor hydroxycamptothecin (HCPT) with a selenium analog of the histone deacetylase (HDAC) inhibitor suberoylanilide hydroxamic acid. SeSA-HCPT showed markedly higher cytotoxicity in prostate cancer (PCa) cells than single or combined treatments, while sparing normal keratinocytes. At effective concentrations, it triggered pronounced S-phase arrest and apoptosis, driven by Topo I inhibition and extensive DNA double-strand breaks; concurrently, SeSA-HCPT suppressed homologous recombination through downregulation of KIF4A and impaired RAD51 recruitment. In a PC-3 xenograft model, SeSA-HCPT significantly inhibited tumor growth relative to the combination treatment without observable systemic toxicity. These results nominate SeSA-HCPT as a promising dual-mechanism therapeutic candidate for advanced PCa.

## Highlights

• SeSA-HCPT is a dual-target inhibitor integrating Topo I and HDAC inhibition
• SeSA-HCPT induces potent DNA damage and S-phase arrest in prostate cancer cells
• SeSA-HCPT impairs homologous recombination by suppressing KIF4A-RAD51 signaling
• Oral SeSA-HCPT markedly inhibits CRPC tumor growth with minimal systemic toxicity

## Graphical abstract

**Figure:**
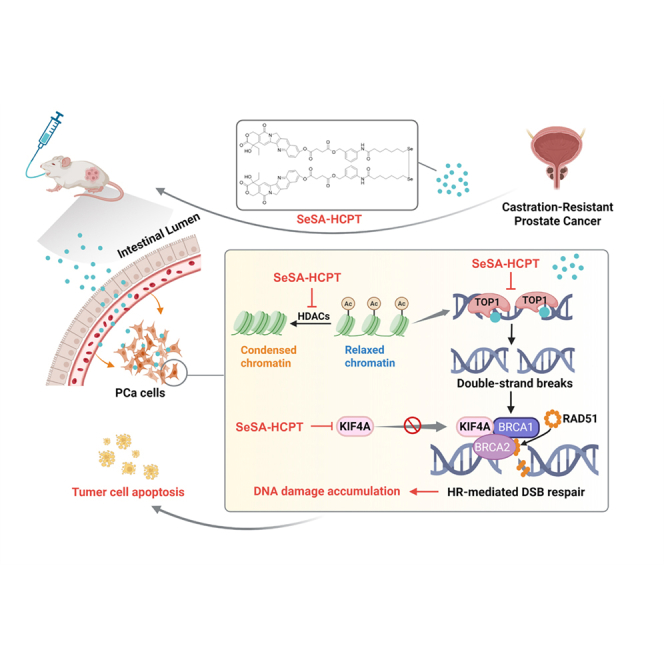

## Introduction

Prostate cancer (PCa), the most common malignancy in men, accounts for approximately 7.3% of new cancer cases globally and ranks as the second leading cause of cancer-related deaths among men.^1^ Castration-resistant prostate cancer (CRPC) is an advanced stage of PCa that progresses despite androgen deprivation therapy (ADT), the standard first-line treatment.^2^ While initially effective, most patients eventually develop CRPC, which is associated with high recurrence rates, poor prognosis, and limited treatment options. Although recent advancements in therapies, such as novel androgen receptor (AR) inhibitors and chemotherapy, have improved outcomes for some patients, significant challenges remain due to tumor heterogeneity and treatment resistance.^3^ This highlights the urgent need for new and more effective treatment strategies to improve the clinical management of CRPC. One promising therapeutic approach is the combination of topoisomerase inhibitors and histone deacetylase (HDAC) inhibitors.

DNA topoisomerases are crucial enzymes that alleviate supercoiling during DNA replication and transcription by cleaving, rotating, and rejoining DNA strands. Topoisomerase inhibitors disrupt this process by preventing the re-ligation of DNA strands, leading to the accumulation of DNA double-strand breaks. These breaks are particularly lethal to rapidly dividing cancer cells, triggering cell-cycle arrest and apoptosis.^4^ Hydroxycamptothecin (HCPT) specifically targets topoisomerase I (Topo1) by stabilizing the Topo1-DNA cleavage complex, effectively blocking DNA re-ligation and causing lethal damage to DNA.^4^^,^^5^ Clinically, HCPT is used to treat various cancers, including gastric, liver, and head and neck cancers, as well as leukemia.^6^ Compared with that in normal tissues, the overexpression of Topo1 in cancer cells makes Topo1 an ideal therapeutic target, increasing the selective anticancer potential of HCPT. However, despite its promising selectivity, the clinical application of HCPT is significantly limited by challenges such as mammalian cell toxicity, lactone ring instability, drug resistance, and poor water solubility.^5^ The development of HCPT prodrugs offers a highly effective strategy to overcome these limitations, enhancing their therapeutic efficacy in cancer treatment.

HDACs are a family of enzymes that play a crucial role in regulating gene expression and various cellular processes. They are classified into four main groups based on their homology and subcellular localization: class I (HDACs 1, 2, 3, and 8), class II (HDACs 4, 5, 6, 7, 9, and 10), class III (SIRT proteins 1–7), and class IV (HDAC11). While most HDACs are zinc-dependent, class III enzymes uniquely rely on NAD+ for their activity.^7^ Over the past two decades, significant progress has been made in the development of HDAC inhibitors, with six currently approved for clinical use.^8^ These inhibitors function by binding to the active site of HDACs, where their hydroxamic acid group chelates the zinc ion essential for enzymatic activity. This inhibition prevents the deacetylation of histones and other substrates, thereby altering gene expression. Vorinostat (suberoylanilide hydroxamic acid, SAHA) was the first HDAC inhibitor approved by the FDA in 2006 for the treatment of cutaneous T cell lymphoma.^9^ While HDACs are frequently overexpressed in various tumors, clinical use of SAHA remains primarily limited to hematological and lymphatic malignancies. Its efficacy in solid tumors is hindered by poor selectivity, high toxicity, and the development of tumor resistance.^10^^,^^11^^,^^12^

Recent research has highlighted the potential of dual-target inhibitors of HDACs and topoisomerase I (Topo1) as effective agents for blocking cancer cell proliferation. For example, Yu et al. synthesized the small molecule WJ35435 by combining the structures of DACA (N-[2-(dimethylamino)ethyl]acridine-4-carboxamide), a Topo1/Topo2 inhibitor, and SAHA, resulting in a compound that exhibits stronger HDAC inhibitory activity than SAHA.^13^ In CRPC cell lines PC3 and DU145, WJ35435 demonstrated superior antiproliferative effects compared with those of both SAHA and DACA. Additionally, He’s research group developed multitarget inhibitors of TOP1/TOP2 and HDAC based on 3-amino-10-HCPT and SAHA, which showed significant antiproliferative and proapoptotic effects.^14^ Cincinelli et al. further contributed to this field by designing dual inhibitors targeting both Topo1 and HDAC, revealing broad antiproliferative activity against various human solid tumors, hematological malignancies, and mesothelioma cell lines, with IC_50_ values in the nanomolar range.^15^ Notably, compared with SAHA or irinotecan (a Topo1 inhibitor) alone, this compound exhibited enhanced antitumor activity in nude mice with orthotopic tumor transplants. Collectively, these findings suggest that developing drugs that simultaneously target HDAC and Topo1 represents a promising strategy for cancer therapy.

In this study, the development of a dual-target approach that combines the HDAC inhibitor SeSAHA (a selenium-containing derivative of SAHA) with the Topo1 inhibitor HCPT into a single “two-in-one” compound, SeSA-HCPT. By simultaneously targeting HDAC and Topo1, SeSA-HCPT synergistically induced DNA damage while inhibiting DNA repair capacity, thereby significantly improving cell death rates in CRPC cell lines compared with the corresponding physical combinations of the two agents. Our findings suggest that SeSA-HCPT has the potential to provide therapeutic benefit in advanced PCa.

## Results

### Synthesis, characterization, and cytotoxicity of SeSA-HCPT

**Figure 1:**
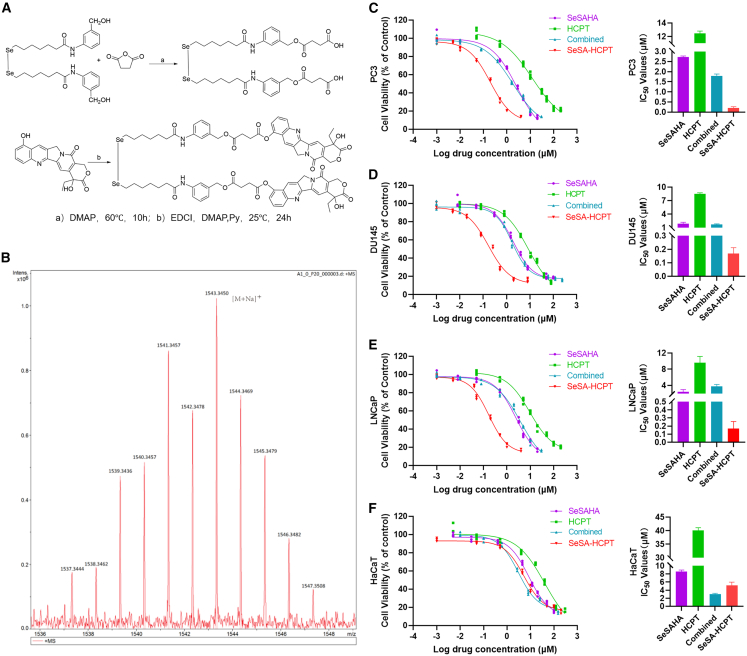
Synthesis and characterization of SeSA-HCPT (A) The synthetic route of SeSA-HCPT. (B) MS spectrum of SeSA-HCPT. (C–F) Cell lines were treated with a range of drug concentrations to assess the cytotoxic activity of SeSA-HCPT, SeSAHA, or HCPT alone or in combination. The cell lines were exposed to drugs for 48 h. A total of 20 μL of MTT reagent was added to each well, and absorbance measurements were taken at 490 nm. All values are averages of replicates expressed relative to cell viability values in untreated cells normalized to 100%. Cytotoxicity curves represent *n* = 3 experiments with 4 replicates per drug concentration for each experiment. Bar graphs (right plots in each panel) show the IC_50_ value of *n* = 3 independent experiments. Standard deviations, SDs, are shown for each drug concentration (left plots) and for each IC_50_ (right plots).

The SeSA-HCPT conjugate was synthesized using the following two-step procedure depicted in Figure 1A. The chemical structures of the intermediates (compound 1 and SeSAHA) and the final product were confirmed by ^1^H NMR, ^13^C NMR, and mass spectrometry (Figures S1, S2, and 1B). To assess the spectrum of antitumor activity, we conducted an initial IC_50_ screening across a panel of tumor cell lines (Table S1). These results indicated that PCa cells were particularly sensitive to SeSA-HCPT: in the tested PCa lines (PC3 and DU145), SeSA-HCPT exhibited a significantly lower IC_50_ than either the individual drugs or their physical combination (Figures 1C and 1D). In contrast, in the non-prostate tumor cell lines included in the screen, SeSA-HCPT and the combination therapy produced similar or higher IC_50_ values, highlighting the relative selectivity of SeSA-HCPT for PCa.

Given that a substantial proportion of advanced PCas are AR-positive, we further evaluate its effects in LNCaP cells, an AR-positive and androgen-sensitive PCa line. Consistent with the findings in AR-negative CRPC models (PC3 and DU145), SeSA-HCPT exhibited a significantly lower IC50 value in LNCaP cells compared with SAHA, HCPT, or their physical combination (Figure 1E). Notablvvy, normal human keratinocyte HaCaT cells were not sensitive to monotherapy, the physical combination of individual drugs, or SeSA-HCPT treatment (Figure 1F). These results suggest that SeSA-HCPT exerts superior anticancer activity across different AR statuses in PCa, while causing minimal damage to normal tissues in patients.

### Effects of SeSA-HCPT on cell cycle progression and apoptosis of PCa cells

Compared with combination therapy, SeSA-HCPT had approximately 10-fold lower IC50, indicating its greater efficacy. To investigate the mechanism of action of SeSA-HCPT in PCa, we examined its effects on cell cycle progression and apoptosis and compared both SeSA-HCPT alone and in combination therapy at equivalent concentrations.

**Figure 2:**
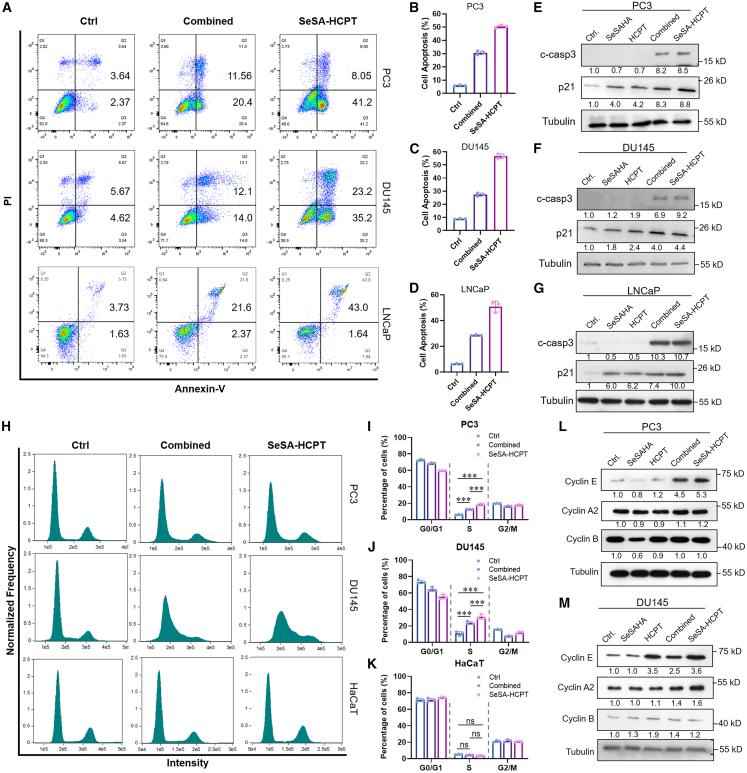
SeSA-HCPT induces S-phase cell-cycle arrest and apoptosis in PCa cells Cells were treated with 200 nM SeSA-HCPT, or combined agents (200 nM SeSAHA +400 nM HCPT). DMSO used as control. (A–D) Apoptosis induction in PCa cell lines by the indicated treatments, as determined by flow cytometry. (E–G) Western blot analysis of cleaved caspase-3 levels in PC3, DU145, and LNCaP cells treated with SeSAHA, HCPT, combination therapy, or SeSA-HCPT. (H) Cell cycle distribution analysis of PC3, DU145, and HaCaT cells treated with either SeSA-HCPT alone or the combination therapy. Analysis was performed using flow cytometry. (I–K) Cell-cycle arrest of PC3, DU145, and HaCaT cells by the tested compounds. (L and M) Western blot analysis of cell cycle-related protein expression in PC3 and DU145 cells treated with SeSAHA, HCPT, combination therapy, or SeSA-HCPT. Data are presented as mean ± SD. Statistical significance is denoted as follows: ∗*p* < 0.05, ∗∗*p* < 0.01, ∗∗∗*p* < 0.001.

We next evaluated apoptosis in PCa cells after 48 h of treatment with SeSA-HCPT at its IC50 concentration, an equimolar combination of the two single agents, or DMSO as a control. Annexin V/PI staining revealed that SeSA-HCPT induced significantly higher levels of apoptosis in PC3, DU145, and LNCaP cells compared with the combination therapy at equivalent concentrations (Figures 2A–2D). Western blot analysis further demonstrated increased levels of cleaved caspase-3 and p21, both of which are key markers of apoptosis activation and cell cycle regulation (Figures 2E–2G). These results demonstrated that SeSA-HCPT is broadly effective against tumors, regardless of their AR status.

Flow cytometry analysis further demonstrated that SeSA-HCPT treatment at IC_50_ for 24 h induced significant S-phase arrest in PC3 and DU145 cells (Figures 2H–2J), as well as in LNCaP cells (Figure S3), indicating disruption of DNA replication processes. Importantly, neither SeSA-HCPT nor the combination therapy altered cell cycle distribution in non-cancerous HaCaT cells at identical concentrations, highlighting the compound’s cancer-specific cytotoxicity (Figure 2K). Western blot analysis revealed that SeSA-HCPT treatment led to upregulation of Cyclin E, whereas Cyclin A and Cyclin B levels remained largely unchanged (Figures 2L and 2M). This expression pattern is consistent with cell-cycle arrest at the early S phase, indicating that SeSA-HCPT interferes with DNA replication initiation or progression rather than later cell cycle transitions. Notably, at the same treatment concentration, HCPT induced a stronger upregulation of Cyclin E in DU145 cells than in PC3 cells, correlating with the lower IC_50_ value of HCPT in DU145 and likely reflecting greater replication stress or S-phase perturbation in this cell line.

### Inhibition of topoisomerase I activity by SeSA-HCPT *in vitro*

**Figure 3:**
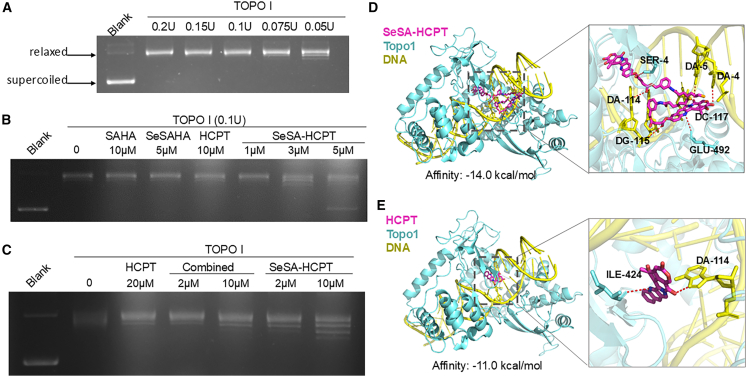
Inhibition of topoisomerase activity of SeSA-HCPT (A) Representative gels showing the results of the Topo1-mediated DNA relaxation assay. pBluescript II supercoiled DNA (0.2 μg) was incubated with decreasing activity units of Topo1 for 30 min at 37 °C. The resolved DNA was stained with GelRed, which revealed distinct bands of supercoiled and relaxed DNA. Lane 1 contains pBluescript II alone, whereas Lanes 2–6 show the effects of progressively reduced Topo1 activity units. (B) pBluescript II supercoiled DNA (0.2 μg) was incubated with Topo1 (0.1 U) alone or Topo1 combined with HCPT, SAHA, SeSAHA, or SeSA-HCPT at the indicated concentrations. (C) pBluescript II supercoiled DNA was incubated with Topo1 alone or with Topo1 plus HCPT, a combined treatment (HCPT and SeSAHA), or SeSA-HCPT at the indicated concentrations. (D and E) Visualization of the binding mode of SeSA-HCPT or HCPT within the Topo1/DNA complex. Hydrogen bonds are depicted as dashed lines, with DNA shown in yellow, Topo1 in blue, and SeSA-HCPT or HCPT in purple.

The accumulation of cells in the S-phase suggests that SeSA-HCPT interferes with DNA replication, a critical vulnerability in rapidly proliferating cancer cells. As a twin drug that combines HCPT and SeSAHA, SeSA-HCPT is hypothesized to impact DNA replication by inhibiting topoisomerase I (Topo1) activity. To evaluate this effect, a Topo1-mediated relaxation assay was performed. This assay relies on the principle that active topoisomerases relax supercoiled DNA, resulting in a Topo1/relaxed DNA complex that migrates more slowly in agarose gel electrophoresis than in supercoiled plasmid DNA. Dose-dependent relaxation was observed when 0.2 μg of pBlueScript II supercoiled DNA was incubated with increasing concentrations of Topo1 (Figure 3A), with as little as 0.1 U of Topo1 being sufficient to completely relax the DNA; this concentration was then used in subsequent experiments.

To assess the effects of SeSA-HCPT, supercoiled DNA was incubated with Topo1 in the presence of HCPT, SAHA, SeSAHA, or SeSA-HCPT. While 10 μM HCPT had no impact on Topo1 activity, SeSA-HCPT at concentrations of 3 and 5 μM effectively inhibited Topo1 activity, as demonstrated by reduced DNA relaxation (Figure 3B). In contrast, SAHA at 10 μM and SeSAHA at 5 μM showed no significant inhibition, indicating that SAHA and SeSAHA do not independently affect Topo1 activity.

To determine whether the enhanced Topo1 inhibition by SeSA-HCPT is associated with the SeSAHA moiety, we further compared the Topo1 inhibitory capacity of SeSA-HCPT with that of a physical combination of HCPT and SeSAHA. As shown in Figure 3C, SeSA-HCPT had the strongest inhibitory effect on Topo1 activity at 10 μM, outperforming the equivalent concentrations of the combined treatment. Notably, the combined treatment group exhibited inhibitory effects similar to those of HCPT alone, indicating that SeSAHA does not enhance the Topo1 inhibitory activity of HCPT *in vitro* (Figure 3C).

Molecular docking studies further revealed that SeSA-HCPT has a significantly greater binding affinity for the Topo1-DNA complex than HCPT alone, likely due to additional hydrogen bonds and other interactions provided by the SeSAHA moiety (Figure 3D). This enhanced binding stabilizes the inhibitor-enzyme-DNA complex, effectively blocking the catalytic function of Topo1 and reducing its ability to relax supercoiled DNA. The experimental data showing reduced DNA relaxation at lower concentrations of SeSA-HCPT align well with the docking predictions, reinforcing the conclusion that increased binding affinity is a key determinant of its superior inhibitory potency.

### Inhibition of HDAC activity by SeSA-HCPT in PCa cells

SAHA is a pan-HDAC inhibitor that specifically targets zinc-dependent HDACs from classes I, II, and IV while leaving the NAD(+)-dependent class III enzymes unaffected. By binding to the zinc ion through its hydroxamic acid group, SAHA inhibits the activity of these HDACs, leading to the suppression of histone and substrate deacetylation. HDAC1-3 and HDAC7 primarily target lysine residues on histones H1, H2A, H2B, and H3, resulting in chromatin condensation and transcriptional repression.^16^^,^^17^ Notably, HDAC4 and HDAC9 exhibit very weak deacetylase activity on histones, whereas HDAC6 primarily deacetylates α-tubulin, influencing microtubule stability.^18^^,^^19^^,^^20^ Therefore, the levels of acetylated histone H3 and acetylated α-tubulin were evaluated as indicators of HDAC activity.

**Figure 4:**
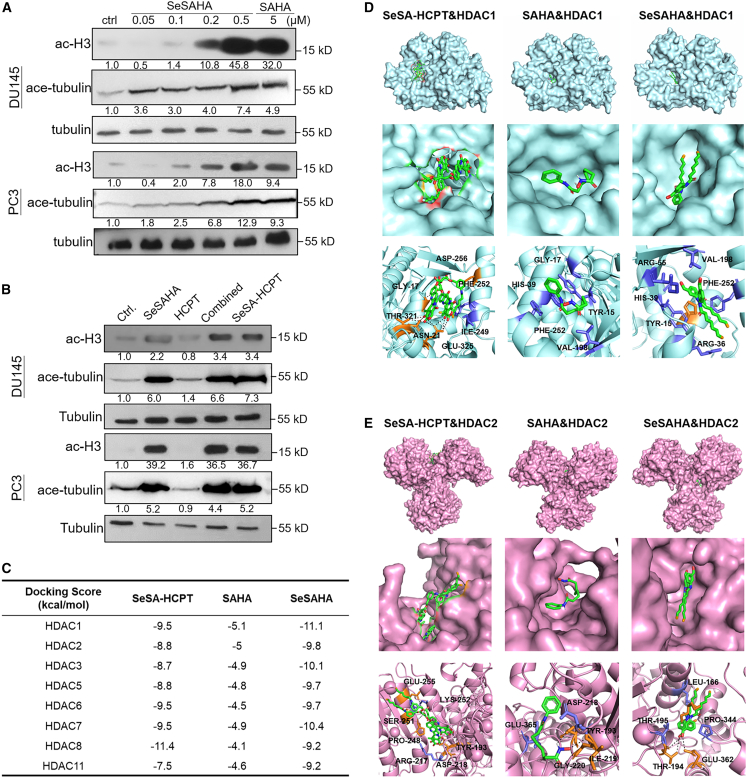
Inhibition of HDAC activity by SeSA-HCPT (A) PCa cells were treated with 5 μM SAHA or varying doses of SeSAHA for 24 h. Whole-cell lysates were prepared and analyzed by western blotting using antibodies against acetylated histone H3 and acetylated tubulin. (B) Cells were cultured with SeSA-HCPT (200 nM) or mono-/combined agents (equimolar concentrations) for 24 h. HDAC inhibitory activity was assessed via western blot analysis. (C) Molecular docking results of HDACs with SeSA-HCPT, SAHA, or SeSAHA. (D and E) Transparent surface view images and active site binding diagrams of HDAC1 (D) and HDAC2 (E) complexed with SeSA-HCPT, SAHA, or SeSAHA. The panels display both the overall active site pocket topology and the key binding interactions, with orange indicating the positions of hydrogen bond formation.

We first compared the inhibitory effects of SeSAHA and SAHA on HDAC activity in PCa cells. After the cells were treated with 5 μM SAHA or 0.05–0.5 μM SeSAHA, we assessed the expression levels of ac-H3 and ace-tubulin, which are markers of HDAC activity. Consistent with previous findings,^21^^,^^22^ SeSAHA resulted in greater inhibition of HDAC activity in PCa cells than SAHA (Figure 4A). Next, we examined the inhibitory effects of SeSA-HCPT and its mono- and combination therapy counterparts on HDAC activity in PCa cells. SeSA-HCPT significantly reduced HDAC activity compared with untreated controls, exhibiting an effect comparable to that of the combination treatment but lower than that of SeSAHA alone (Figure 4B).

To explore the underlying mechanism, molecular docking studies were conducted to assess the binding interactions of SeSA-HCPT, SAHA, and SeSAHA with various HDAC isoforms. Overall, both SeSA-HCPT and SeSAHA, which exhibit equivalent HDAC inhibitory activity, showed significantly stronger predicted binding affinities than SAHA, which may be partially attributed to their stoichiometric differences (Figure 4C). However, isoform-specific variations were observed: in several HDAC isoforms, such as HDAC1 and HDAC2, SeSAHA exhibited slightly higher binding scores than SeSA-HCPT, while in HDAC8, SeSA-HCPT not only achieved higher docking scores but also formed a greater number of hydrogen bonds within the active site. In HDAC6, the two compounds showed comparable affinities.

To further elucidate the structural basis underlying these differences, transparent surface models and active site binding diagrams of HDAC1 and HDAC2 complexed with SeSA-HCPT, SAHA, and SeSAHA were generated (Figures 4D and 4E). These models revealed that although SeSAHA fits very well into the active site pocket and establishes favorable hydrogen bonding interactions, SeSA-HCPT—with its additional hydrogen bond interactions in certain cases—does not lead to any enhancement in overall binding affinity relative to SeSAHA. In contrast, additional PyMOL analyses of HDAC8, including both transparent surface views and detailed active site diagrams, revealed that the increased number of hydrogen bonds formed by SeSA-HCPT led to a greater binding affinity in this isoform than in SeSAHA (Figure S4).

These results suggest that the HDAC inhibitory activity of SeSA-HCPT is largely attributable to its SeSAHA moiety and that the addition of HCPT does not further improve its binding to HDACs. Consequently, comparable HDAC inhibition by SeSA-HCPT and SeSAHA led to similar degrees of chromatin relaxation. This chromatin remodeling likely facilitates enhanced DNA accessibility for the HCPT moiety, thereby contributing to the overall anticancer efficacy of SeSA-HCPT in PCa cells without altering HDAC inhibition per se.

### Low concentrations of SeSA-HCPT effectively induce DNA damage and inhibit growth and migration of PCa cells

**Figure 5:**
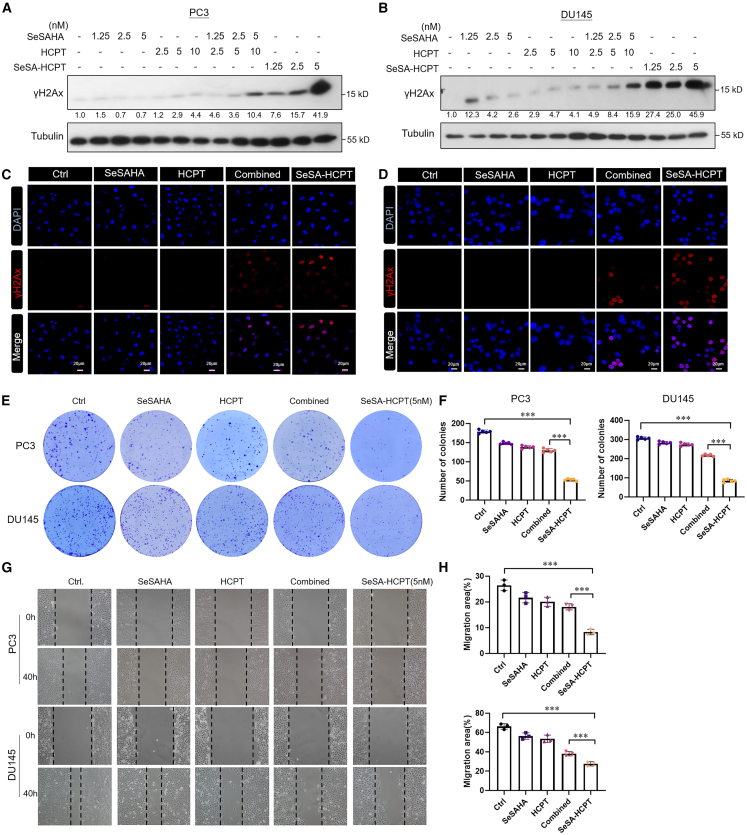
SeSA-HCPT exhibits superior anticancer effects at low concentration in PCa cells (A and B) Western blot analysis of γ-H2AX levels in PC3 and DU145 cells subjected to various drug treatments or control condition at the indicated concentrations. (C–H) Cells were treated with 5 nM SeSA-HCPT, combined agents (5 nM SeSAHA +10 nM HCPT), 5 nM SeSAHA, or 10 nM HCPT. DMSO used as control. Immunofluorescence staining for γ-H2AX (red) in PC3 and DU145 cells treated with drugs, with nuclei counterstained using DAPI (blue). Scale bars, 20 μm (C and D). Colony formation assays were performed to evaluate the proliferation capacity of PC3 and DU145 cells under the same treatment conditions (E). Quantification of the proliferation capacity of PC3 and DU145 cells based on the colony formation assay (F). The migratory capacity of PC3 and DU145 cells was assessed using a wound healing assay (G). Wound closure was quantified using ImageJ software (H). Data are presented as mean ± standard deviation, with statistical significance indicated as follows: ∗*p* < 0.05, ∗∗*p* < 0.01, and ∗∗∗*p* < 0.001.

SeSA-HCPT demonstrated superior topoisomerase I (Topo1) inhibitory activity, prompting screening of various concentrations to identify the effective dose for inducing DNA damage in PCa cells, as assessed by γ-H2AX expression. PCa cells were treated with a range of SeSA-HCPT concentrations, reference drugs (both individually and in combination at equimolar concentrations), and DMSO. Western blot analysis revealed a dose-dependent increase in γ-H2AX levels following treatment with both SeSA-HCPT and the combination therapy at lower concentrations (1.25–5 nM) in both PC3 and DU145 cells, indicating significant DNA damage (Figures 5A and 5B). Notably, this effect was more pronounced with SeSA-HCPT than with the combination therapy at the same concentrations. Immunofluorescence staining (Figures 5C and 5D) further confirmed the increase in γ-H2AX expression in SeSA-HCPT-treated cells, with strong red fluorescence localized to the nuclei of PC3 and DU145 cells. These findings suggest that SeSA-HCPT effectively induces DNA damage at nanomolar concentrations, resulting in higher γ-H2AX expression than that resulting from the combination therapy.

To further validate the anticancer activity of SeSA-HCPT at these concentrations, we assessed its effects on cell growth and migration. A colony formation assay indicated that treatment with 5 nM SeSA-HCPT significantly inhibited the proliferation of both PC3 and DU145 cell lines, resulting in markedly stronger inhibitory effects than those observed with HCPT, SeSAHA, or their combination at the same concentration (Figure 5E). Compared with the control treatment, SeSA-HCPT treatment reduced colony formation by more than 70.4 ± 2.89% in PC3 cells and by approximately 76.5 ± 3.73% in DU145 cells (Figure 5F). Additionally, wound healing assays demonstrated that 5 nM SeSA-HCPT substantially decreased the migration rates of both PC3 and DU145 cells (Figures 5G and 5H). In contrast, neither HCPT nor SeSAHA alone or their combination had significant inhibitory effects at this concentration (Figures 5G and 5H). Compared with the other treatments, SeSA-HCPT treatment reduced the migration rates of both cell lines by more than 10%. The high potency of SeSA-HCPT at low concentrations indicates the potential for reduced dosing regimens, which could minimize side effects while preserving therapeutic efficacy.

### Enhanced cytotoxicity of SeSA-HCPT through synergistic induction of DNA damage and inhibition of DNA repair mechanisms

**Figure 6:**
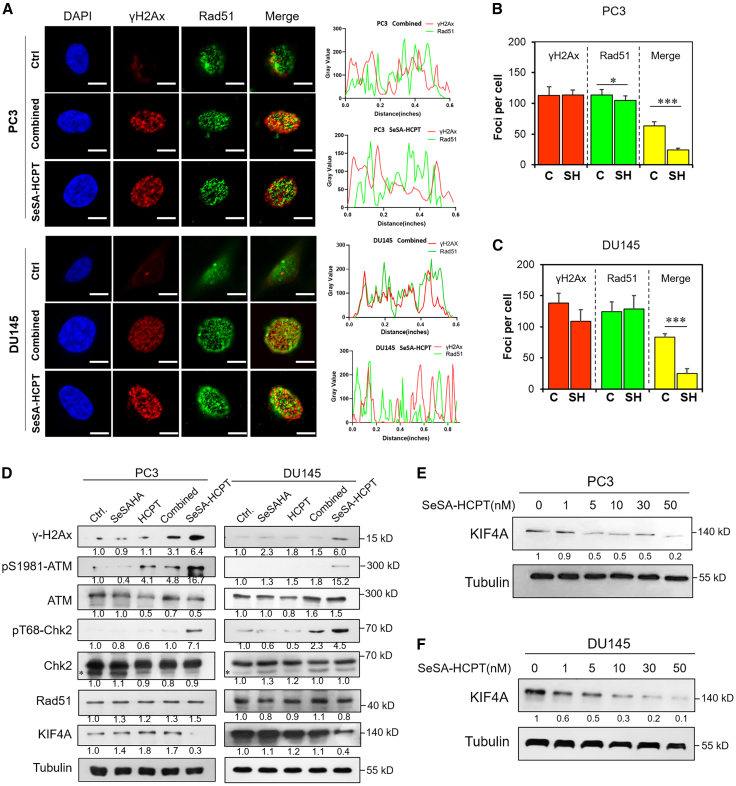
SeSA-HCPT impedes the recruitment of Rad51 to the site of damage by decreasing KIF4A expression (A–D) Cells were treated with 5 nM SeSA-HCPT, combined agents (5 nM SeSAHA +10 nM HCPT), 5 nM SeSAHA, or 10 nM HCPT. DMSO used as control. (A) Immunofluorescence staining showing the co-localization of γ-H2AX (red) and Rad51 (green) in PC3 and DU145 cells treated with 5 nM SeSA-HCPT or equivalent combination for 24 h. Scale bars, 10 μm (B and C) Histogram quantification of γ-H2AX and Rad51 puncta per cell (*n* = 50). (D) Western blot analysis of γ-H2AX, pS1981-ATM, ATM, pT68-Chk2, Chk2, Rad51, and KIF4A levels in PC3 and DU145 cells after indicated drug treatments. (E and F) Western blot analysis showing KIF4A levels in PC3 and DU145 cells treated with SeSA-HCPT at the indicated concentrations for 24 h. All the data are expressed as the means ± SDs from at least three independent experiments. ∗*p* < 0.05, ∗∗*p* < 0.01, ∗∗∗*p* < 0.001.

Both SeSA-HCPT and the combination therapy induced significant DNA damage. However, at equivalent concentrations, SeSA-HCPT treatment elicited higher levels of apoptosis, suggesting that it may involve additional mechanisms beyond DNA damage. We hypothesized that SeSA-HCPT might compromise cellular DNA damage repair capacity. To investigate this possibility, we examined Rad51 expression and its nuclear co-localization with γ-H2AX following treatment with either 5 nM SeSA-HCPT or combination therapy. Immunofluorescence analysis revealed that SeSA-HCPT significantly suppressed the co-localization of γ-H2AX and Rad51 compared to that of combination treatment in both PC3 and DU145 cells (Figure 6A). Quantitative analysis demonstrated a significant increase in the number of γ-H2AX foci per cell following both SeSA-HCPT and combination treatments, confirming substantial induction of DNA damage (Figures 6B and 6C). Importantly, cells treated with SeSA-HCPT exhibited markedly reduced Rad51 recruitment to damage sites compared with that of combination treatment, indicating that SeSA-HCPT not only induces DNA damage but also impairs Rad51-mediated DNA repair processes.

Western blot analysis further corroborated these findings by revealing elevated levels of γ-H2AX and phosphorylated ATM (pS1981-ATM) following treatment with either SeSA-HCPT or the combination therapy, indicating an active DNA damage response (Figure 6D). Notably, while both treatments had no effect on Rad51 expression, SeSA-HCPT treatment markedly downregulated KIF4A, a protein known to associate with BRCA2 and facilitate Rad51 recruitment during homologous recombination (HR) repair.^23^^,^^24^ Subsequent dose-response studies indicated that SeSA-HCPT treatment induced a dose-dependent decrease in KIF4A levels in both PC3 and DU145 cells, providing a potential mechanistic explanation for the observed impairment of DNA repair (Figures 6E and 6F). Collectively, these results suggest that the superior efficacy of SeSA-HCPT in killing cancer cells compared with that of combination therapy arises from its dual action; that is, inducing DNA damage while concurrently inhibiting DNA repair through the downregulation of KIF4A, thereby impairing Rad51 recruitment.

### SeSA-HCPT inhibits tumor growth through induction of DNA damage *in vivo*

**Figure 7:**
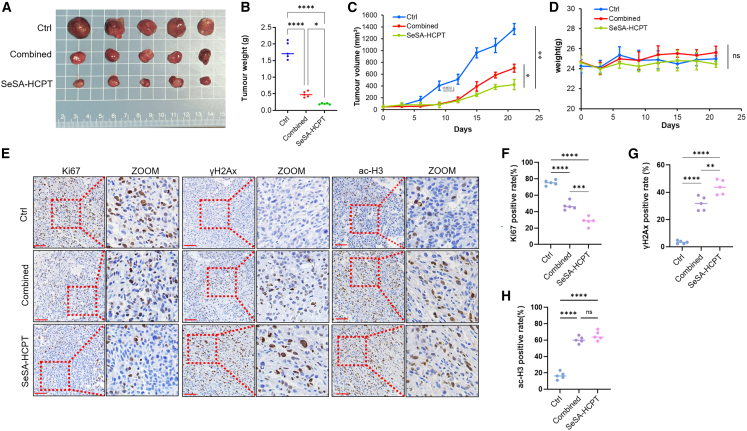
Effectiveness of SeSA-HCPT in inhibiting tumor growth (A) Representative images of tumors excised from nude mice injected subcutaneously with PC3 cells following gavage administration of SeSA-HCPT or combined treatment. (B) Statistical analysis of tumor weights with a sample size of *n* = 5. (C) Tumor growth curves corresponding to the treatments. (D) Body weight curves of PC3 tumor-bearing mice subjected to different treatments. (E) Immunohistochemical staining for Ki67 and γ-H2AX in tumors from the mice. (F and G) Quantification of Ki67-positive or γ-H2AX-positive cells from the histograms. Scale bars, 100 μM. Statistical significance: ∗∗*p* < 0.01, ∗∗∗*p* < 0.001, ∗∗∗∗*p* < 0.0001.

The antitumor efficacy of SeSA-HCPT was evaluated in PC3 xenograft-bearing nude mice. After 21 days of treatment, tumor specimens were collected and analyzed (Figure 7A). SeSA-HCPT treatment resulted in significantly reduced tumor weight compared with that in both the control (*p* < 0.01) and HCPT+SeSAHA physical mixture groups (*p* < 0.05; Figure 7B). Tumor volume measurements throughout the treatment period consistently demonstrated superior growth suppression in the SeSA-HCPT group compared with that in both control and physical mixture groups (*p* < 0.01 and *p* < 0.05, respectively) (Figure 7C). No significant differences in body weight were observed among the three groups during the study period (Figure 7D). Furthermore, serum biochemical analysis revealed no significant alterations in hepatic (ALT, AST, and ALP) or renal (BUN and creatinine) parameters in SeSA-HCPT-treated mice relative to controls (Table 1), confirming low systemic toxicity of SeSA-HCPT.

**Table 1:** Serum biochemical parameters of nude mice after drug administration

Parameters	DMSO	HCPT+SeSAHA	SeSA-HCPT
ALT (U/L)	32.4 ± 4.1	34.3 ± 3.8	34.8 ± 4.2
AST (U/L)	279.4 ± 36.1	294.5 ± 35.7	283.1 ± 27.4
ALP (U/L)	48.2 ± 3.4	53.8 ± 3.6	50.4 ± 4.0
BUN (mmol/L)	7.8 ± 0.6	8.2 ± 0.7	8.0 ± 1.0
Creatinine (μmol/L)	12.1 ± 3.2	13.5 ± 2.8	12.0 ± 3.5

Immunohistochemical analysis revealed significant decreases in Ki67-positive cells in both treatment groups, with SeSA-HCPT showing the most pronounced reduction compared with that in the control (*p* < 0.001) and physical mixture (*p* < 0.01) groups (Figures 7E and 7F). Conversely, γ-H2AX immunostaining revealed progressively increased DNA damage markers, with SeSA-HCPT inducing significantly higher levels than the control (*p* < 0.001) and physical mixture (*p* < 0.05) groups (Figure 7G). Notably, SeSA-HCPT was administered via oral gavage, offering a practical advantage over the conventional intravenous delivery required for HCPT. These results indicate that SeSA-HCPT exerts potent antitumor activity through DNA damage-mediated growth inhibition.

## Discussion

Chemotherapy remains a cornerstone of cancer treatment; however, single-agent therapies frequently encounter limitations such as suboptimal efficacy, the development of drug resistance, and severe side effects. To overcome these challenges, combination therapies have become increasingly popular in clinical practice. However, traditional two-drug combinations often require separate dosing schedules, potentially complicating treatment regimens, increasing the risk of drug-drug interactions, and resulting in variable pharmacokinetic profiles.^25^ These factors can hinder the achievement of optimal therapeutic levels and consistent drug exposure in patients.

In this study, we developed a novel twin drug, SeSA-HCPT, that integrates the DNA-damaging properties of HCPT, a topoisomerase I inhibitor, with the epigenetic regulatory activity of SAHA, a histone deacetylase inhibitor (HDACi). SeSA-HCPT exhibited significantly greater cytotoxicity in both AR-positive and AR-negative PCa cells than the combination of HCPT and SeSAHA, underscoring the therapeutic advantage of its dual-targeting design. These findings suggest that the two-in-one formulation of SeSA-HCPT offers a more streamlined and potentially more effective therapeutic strategy for CRPC.

Mechanistically, cellular HDAC activity assays indicate that SeSA-HCPT does not acquire additional HDAC inhibitory potency from the HCPT moiety; its HDAC inhibition is derived solely from the SeSAHA portion, a finding further supported by molecular docking. In contrast, biochemical Topo I assays demonstrate that SeSA-HCPT directly suppresses Topo I activity more effectively than HCPT or the physical combination, consistent with docking predictions indicating a more stable Topo I–DNA complex. In cellular models, SeSA-HCPT induces DNA damage more potently than the combination treatment. The enhanced effects on cell proliferation, apoptosis, and DNA damage observed with SeSA-HCPT are therefore primarily attributed to its superior inhibition of Topo I and the resulting increase in DNA damage.

Resistance to DNA-damaging agents is often linked to the activation of various DNA repair pathways that allow cancer cells to resolve therapy-induced lesions.^26^^,^^27^ Clinically, combining DNA-damaging agents with DNA repair inhibitors has shown promise in preventing the repair of toxic DNA lesions and thereby, enhancing cell death.^28^^,^^29^^,^^30^ Our results suggest that SeSA-HCPT not only induces DNA damage by inhibiting Topo I but also disrupts DNA repair by downregulating KIF4A, a key factor involved in recruiting RAD51 to sites of DNA damage. This impairment of HR further sensitizes cancer cells to treatment. However, alternative factors within the HR pathway may potentially compensate for the impaired repair function, which warrants further investigation.

SeSA-HCPT appears to reduce KIF4A levels through multiple mechanisms. One possibility is that it promotes the degradation of the KIF4A protein via the activation of E3 ubiquitin ligases, thereby targeting KIF4A for proteasomal degradation.^31^ Alternatively, by inhibiting Topo1, SeSA-HCPT may downregulate the transcription of KIF4A, interfering with essential DNA unwinding processes necessary for gene expression. In addition, acetylation prediction analyses (using http://pail.biocuckoo.org/) have identified several potential acetylation sites on KIF4A, suggesting that post-translational modifications may also regulate its stability and function.^32^ These modifications, however, warrant further investigation.

Although HCPT and SAHA are not established as standard treatments for CRPC, our study reveals that their integration into the single molecule SeSA-HCPT has exceptional therapeutic efficacy against this aggressive cancer. This enhanced performance can be attributed to two key factors: (1) mechanistic synergy: HCPT targets DNA replication by stabilizing the Topo1-DNA complex, whereas the SeSAHA component promotes histone hyperacetylation, increasing chromatin accessibility. This dual action creates a synergistic environment in which cells become significantly more vulnerable to DNA damage.^33^ (2) Selenium’s therapeutic contribution: the incorporation of selenium into SeSA-DCA leverages its redox-sensitive release to generate tumor-specific reactive oxygen species (ROS), thereby amplifying the selective activity of both HDAC inhibitors and DCA. This targeted mechanism induces cell-cycle arrest, downregulates CDC25A, and triggers apoptosis, resulting in significant suppression of PCa cell proliferation, invasion, and metastasis, as demonstrated in our previous study.^21^

**Figure 8:**
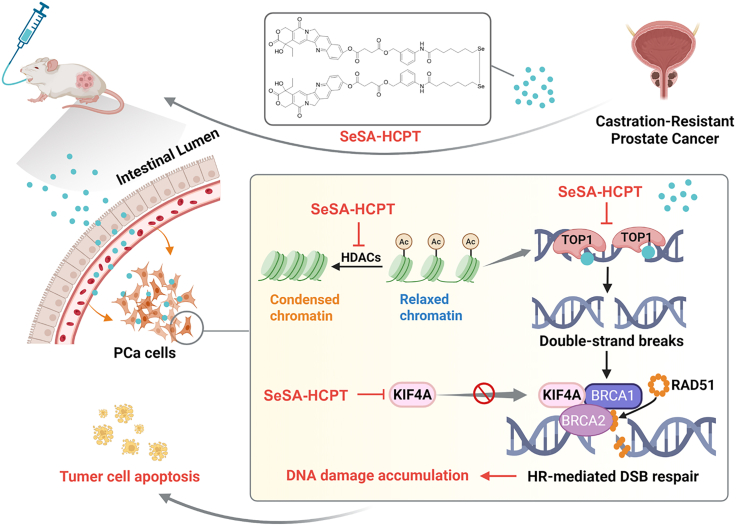
Proposed model outlining the potential anticancer molecular mechanisms of SeSA-HCPT

In conclusion, our study demonstrates that integrating a Topo1 inhibitor with an HDAC inhibitor into a single formulation not only amplifies DNA damage but also simultaneously impairs DNA repair pathways, thereby markedly enhancing therapeutic efficacy in CRPC (Figure 8). This dual-targeting approach represents a promising new strategy for overcoming drug resistance in advanced PCa and may pave the way for the development of more effective therapies in the future.

### Limitations of the study

This study is limited by the use of a single xenograft model, which may not fully capture the heterogeneity of CRPC, and by the lack of comprehensive pharmacokinetic and long-term toxicity analyses. In addition, although KIF4A downregulation was identified as a key mechanism underlying impaired HR, the precise molecular pathways regulating KIF4A expression and stability in response to SeSA-HCPT remain to be fully elucidated.

## Resource availability

### Lead contact

Requests for further information and resources should be directed to and will be fulfilled by the lead contact, Changjun Zhu (skyzcj@tjnu.edu.cn).

### Materials availability

All data are available in the manuscript text and supplemental information. Reagents generated in this study will be made available on request, but we may require a payment and/or a completed Materials Transfer Agreement if there is potential for commercial application.

### Data and code availability

• Data reported in this paper will be shared by the lead contact upon request.
• This paper does not report original code.
• Any additional information required to reanalyze the data reported in this paper is available from the lead contact upon request.

## Acknowledgments

This study was supported by grants from the 10.13039/501100001809National Natural Science Foundation of China (81703013) and the 10.13039/501100006606Natural Science Foundation of Tianjin (23JCYBJC01730). The authors acknowledge American Journal Experts (AJE) for English language editing assistance.

## Author contributions

W. Yajie, conceptualization, methodology, writing – original draft preparation; W.Q. and M.L., data curation, investigation; W.X., data curation; L.X. and Z.T., software and visualization; W.Yuqing and W.S., software and validation; W. Yanming and Z.C., writing – reviewing and editing, supervision, and funding acquisition.

## Declaration of interests

No other conflict of interest exists between the authors and this article.

## STAR★Methods

### Key resources table

**Table:** 

REAGENT or RESOURCE	SOURCE	IDENTIFIER
**Antibodies**		

anti-γH2AX	Epitomics	Cat# ab81299
anti-ac-Histone H3	Huabio, China	Cat# ET1609-8
anti-ace-tubulin	Sigma-Aldrich	Cat# T7451
anti-Cyclin A2	Santa Cruz Biotechnology	Cat# sc-53234
anti-ATM	Epitomics	Cat# ab201022
anti-Chk2	Epitomics	Cat# ab109413
anti-Tubulin	Sigma-Aldrich	Cat# T5168
anti-p21	Santa Cruz Biotechnology	Cat# sc-471
anti-Cleaved-casapase3	Affinity bioscience	Cat# AF7022
anti-Rad51	GeneTex	Cat# GTX70230
anti-Cyclin B1	Santa Cruz Biotechnology	Cat# sc-245
anti-Cyclin E	Santa Cruz Biotechnology	Cat# sc-247
anti-*p*-ATM	Epitomics	Cat# ab81292
anti-*p*-Chk2	Epitomics	Cat# ab207446
anti-actin	Sigma-Aldrich	Cat# a2066
anti-Ki67	Servicebio	Cat# GB111499
anti-KIF4A	Santa Cruz Biotechnology	Cat# sc-365144

**Chemicals, reagents and medium**		

Succinic anhydride	Tianjin Bohai Chemical Co.	–
tetrahydrofuran	Tianjin Bohai Chemical Co.	–
dichloromethane	Tianjin Bohai Chemical Co.	–
N,N-dimethylformamide	Tianjin Bohai Chemical Co.	–
4-Dimethylaminopyridine	Shanghai Sine Chemical Co.	–
1-ethyl-3-(3-dimethylaminopropyl)carbodiimide hydrochloride	Shanghai Sine Chemical Co.	–
10-hydroxycamptothecin	Shanghai Sine Chemical Co.	–
dimethyl sulfoxide	Beijing Huaxia Chemical Co.	–
Pyridine	Beijing Huaxia Chemical Co.	–
Fluoromount-G™ mounting medium	Invitrogen (#00-4958-02)	–
RPMI-1640	HyClone	–
DMEM	HyClone	–
PI/RNase staining buffer	Solarbio (#C0080)	–

**Critical commercial assays**		

FITC-Annexin V/PI apoptosis kit	Solarbio	Cat#CA1020
anti-mouse/rabbit universal immunohistochemical detection kit	Proteintech	Cat#PK10006

**Biological samples**		

Mouse tissues and organs	BALB/c nude mice	–

**Experimental models: Animals**		

BALB/c nude mice	Beijing Vital River	–

**Experimental models: Cell lines**		

Bel-7402	The Cell Bank of Chinese Academy of Sciences	Cat#TCHu 81
HCT116	ATCC	Cat#CCL-247
A549	ATCC	Cat# CCL-185
MCF7	ATCC	Cat# HTB-22
MDA-MB-231	ATCC	Cat# HTB-26
U2OS	ATCC	Cat# HTB-96
HeLa	ATCC	Cat# CCL-2
PC3	ATCC	Cat# CRL-1435
DU145	ATCC	Cat# HTB-81
LNCaP	ATCC	Cat# CRL-1740
HaCaT	The Cell Bank of Chinese Academy of Sciences	Cat#GDC0147

**Software and algorithms**		

GraphPad Prism 9	GraphPad Software	https://www.graphpad.com
Chemdraw 19.0	PerkinElmer	perkinelmerinformatics.com/products/research/chemdraw
ImageJ	NIH	https://imagej.nih.gov/ij
FlowJo X	BD Biosciences	https://www.flowjo.com
IDEAS software	Cytek	https://cytekbio.com/search?q=ideas
AutoDock Vina	The Scripps Research Institute	https://vina.scripps.edu

**Others**		

Multimode Microplate Reader	Tecan	Spark
Confocal laser microscope	Nikon	Nikon A1-Storm
Amnis FlowSight Imaging Flow Cytometer	Cytek	https://cytekbio.com/pages/flowsight
FACS Calibur equipment	BD Biosciences	–

### Experimental model and study participant details

#### Animals

Male 5-week-old BALB/c nude mice were purchased from VitalRiver (Beijing, China). All animal experimental procedures were approved by the Animal Care and Use Committee of Tianjin Normal University (No.2022092001), Tianjin, China.

#### Cell lines

The following human cancer cell lines were utilized in this study: prostate cancer cell lines PC3, DU145 and LNCaP; colon cancer cell line HCT116; cervical cancer cell line HeLa; lung adenocarcinoma cell line A549; breast cancer cell lines MCF7 and MDA-MB-231; hepatoma cell line BEL-7402; and osteosarcoma cell line U2OS. Additionally, human normal keratinocytes (HaCaT) were included. Cells were purchased from ATCC or The Cell Bank of Chinese Academy of Sciences, and cultured according to the supplier’s instructions. Based on the quality control report provided by the supplier, the cell line has been authenticated and confirmed free of mycoplasma contamination. All cell lines were routinely tested for mycoplasma contamination using PCR-based assays and were confirmed to be negative.

The PC3, DU145, LNCaP and HCT116 cell lines were cultured in complete RPMI-1640 medium supplemented with 10% fetal bovine serum (FBS) and 1% penicillin/streptomycin. The U2OS, HeLa, MCF7, MDA-MB-231, BEL-7402, A549 cell lines, and normal keratinocytes (HaCaT) were maintained in DMEM also supplemented with 10% FBS and 1% penicillin/streptomycin.

### Method details

#### Synthesis of compound 1

SeSAHA was synthesized according to a previously reported method.^21^ The product was characterized and matched reported specifications. To a solution of DmSeSAHA (100 mg, 0.159 mmol) in anhydrous tetrahydrofuran (THF, 5 mL), succinic anhydride (47.8 mg, 0.478 mmol) and 4-Dimethylaminopyridine (DMAP, 3.89 mg, 0.032 mmol) were added under N_2_ atmosphere. The mixture was refluxed at 66°C for 2 h. After cooling to room temperature, the solvent was removed under vacuum. The residue was dissolved in ethyl acetate (5 mL), washed with brine (3 × 5 mL), and dried over Na_2_SO_4_. The crude product was purified by trituration with dichloromethane (DCM) to give compound 1 as a white solid (84.2 mg, 63.1%). ^1^H NMR (400 MHz, MeOD): δ 7.67–7.37 (*m*, 4H), 7.28 (*t, J* = 7.9 Hz, 2H), 7.08 (*d, J* = 7.6 Hz, 2H), 5.10 (*s*, 4H), 2.91 (*t, J* = 7.3 Hz, 4H), 2.81–2.52 (*m*, 8H), 2.37 (*t, J* = 7.4 Hz, 4H), 1.96–1.57 (*m*, 8H), 1.56–1.34 (*m*, 8H). ^13^C NMR (101 MHz, MeOD): δ = 174.54,173.24,172.56, 138.73, 136.90, 128.57, 123.17, 119.48, 119.25, 65.78, 36.51, 30.40, 29.25, 28.71, 28.48, 28.37, 28.34, 25.32.

#### Synthesis of SeSA-HCPT

To a solution of compound 1 (50.0 mg, 0.060 mmol) in anhydrous N,N-dimethylformamide (DMF, 4 mL), 1-ethyl-3-(3-dimethylaminopropyl)carbodiimide hydrochloride (EDCI·HCl, 26.5 mg, 0.138 mmol) and DMAP (16.0 mg, 0.131 mmol) were added under N_2_ atmosphere. After stirring for 1 h at room temperature, 10-hydroxycamptothecin (HCPT, 47.8 mg, 0.131 mmol) in DMF (1 mL) was added at 0°C, followed by pyridine (32 μL). The mixture was stirred at room temperature for 12 h. The crude product was purified by dialysis against dimethyl sulfoxide (DMSO, 3 × 2 L, 24 h) and then water. Lyophilization gave A1 as a pale-yellow powder (68.0 mg, 74.0% yield). ^1^H NMR (400 MHz, *DMSO-d6*): δ 9.91 (*s*, 2H), 8.65 (*s*, 2H), 8.18 (*d, J* = 9.2 Hz, 2H), 7.80 (*d, J* = 2.2 Hz, 2H), 7.65 (*s*, 2H), 7.54 (*d, J* = 7.0 Hz, 2H), 7.34 (*s*, 2H), 7.27 (*t, J* = 7.8 Hz, 2H), 7.04 (*d, J* = 7.4 Hz, 2H), 6.54 (*s*, 2H), 5.43 (*s*, 4H), 5.28 (*s*, 4H), 5.11 (*s*, 4H), 2.95 (*d, J* = 6.7 Hz, 4H), 2.82 (*dt, J* = 9.4 Hz, 8H), 2.26 (*t, J* = 7.2 Hz, 4H), 1.96–1.78 (*m*, 4H), 1.69–1.50 (*m*, 8H), 1.26 (*d, J* = 6.4 Hz, 8H), 0.88 (*t, J* = 7.2 Hz, 7H). ^13^C NMR (101 MHz, *DMSO-d6*) δ = 172.93, 172.18, 171.76, 171.40, 157.24, 153.03, 150.46, 149.32, 146.34, 145.78, 139.96, 136.93, 131.66, 130.92, 130.85, 129.29, 128.73, 126.29, 123.07, 119.60, 119.53, 119.14, 118.99, 97.18, 72.83, 66.32, 65.71, 50.68, 36.76, 30.74, 30.61, 29.67, 29.41, 29.19, 28.95, 28.57, 25.40, 8.24.

#### Cell cytotoxicity assays

The cytotoxic effects of the dual-target inhibitor SeSA-HCPT, its reference drugs, and combination drugs were evaluated using the MTT assay in various tumor cell lines. Experiments were performed in triplicate, with each cell line seeded at a density of 3 × 10^3^ cells per well in a 96-well plate. After 48 h of incubation, absorbance was measured using a microplate reader at a wavelength of 570 nm to determine cell viability.

#### Wound healing assay

The effect of SeSAHA-HCPT on cell migratory ability was assessed by *in vitro* wound-healing assay. Briefly, PC3 or DU145 cell were seeded at a density of 5 × 10^4^ cells/ml on 6-well plate. After the cells reached 90% confluence, the wound was made across the middle of each well using a sterile 200 μL pipet tip, and the cell debris was washed twice with PBS. The fresh medium supplied with indicated drugs was added to the wells. Scratches were observed and imaged under the microscope after incubation for 0 and 48 h. The wound area was calculated using the ImageJ software.

#### Colony formation assay

The proliferation of cells was analyzed using the colony formation assays. PC3 or DU145 cells were plated in 6-well plates, incubated for 24 h, and then treated with the indicated drugs or DMSO as a control. Culture media was replaced every 3 days. Following 2 weeks of incubation, surviving colonies (>50 cells per colony) were visualized with 0.1% crystal violet staining.

#### Topo I inhibitory effects of SeSA-HCPT

Plasmid relaxation assay was used to determine the effect of compounds on topoisomerase I activity *in vitro*. Briefly, supercoiled plasmids were incubated with topoisomerase I enzyme and the test compounds. The reaction products were then analyzed by agarose gel electrophoresis to visualize the conversion of supercoiled plasmids to relaxed circular forms.

#### Western blot

Cells were lysed using RIPA buffer supplemented with protease inhibitor. A total of 40 μg of the lysates were loaded in SDS-PAGE gel, subjected to gel electrophoresis in a Western blot apparatus and transferred to a PVDF membrane. Densitometric analysis was performed for the quantification of protein bands on the blot using ImageJ.

#### Immunofluorescence

Slides were rinsed three times with 0.1% Triton X-100 in PBS (PBST) and then blocked with a solution of 1% goat serum and 2% BSA in PBS at room temperature for 1 h. Following blocking, the slides were incubated with a primary antibody conjugated to a fluorescent marker. After incubation, the slides were washed three times with PBST and mounted using Fluoromount-G mounting medium. Fluorescence images were captured using a Nikon A1 confocal laser microscope.

#### Cell cycle analysis

Cell cycle distribution was analyzed using flow cytometry. Following drug treatment, cells were harvested and centrifuged at 1,000 rpm for 5 min. The cell pellets were fixed in 70% ethanol at 4 °C for 24 h. After two washing steps to remove the ethanol, cells were resuspended in 0.5 mL of PI/RNase staining buffer and incubated for 15 min at room temperature. Cell cycle phase distribution was determined using an Amnis FlowSight Imaging Flow Cytometer, and data analysis was performed using IDEAS software.

#### Assessment of apoptosis

For apoptosis analysis, cells were seeded in 6-well plates and treated with drugs for 48 h. Apoptotic cells were detected using the FITC-Annexin V/PI apoptosis kit according to the manufacturer’s protocol. Flow cytometric analysis was performed using FACS Calibur equipment, and the resulting data were analyzed using FlowJo X software.

#### Molecular docking

The three-dimensional structures of SeSAHA and SeSA-HCPT were prepared, and energy was minimized using Open Babel software.^34^ The crystal structures of topoisomerase I-DNA (PDB ID: 1A31), HDAC1 (PDB ID: 5ICN), HDAC2 (PDB ID: 4LXZ), HDAC3 (PDB ID: 4A69), HDAC6 (PDB ID: 6VNR), HDAC7 (PDB ID: 3C0Z) and HDAC8 (PDB ID: 1T69) were retrieved from the RCSB Protein DataBank. The 3D structure of HDAC5 (UniProtID: Q9UQL6) and HDAC11 (UniProtID: Q96DB2) predicted with AlphaFold^35^^,^^36^ were downloaded from the UniProt database.^37^ Molecular docking studies were conducted using AutoDock Vina.^38^

#### Animal model and treatments

PC3 cells were washed with PBS twice and injected subcutaneously into nude mice (5 × 10^6^ cells/site). Once the mouse tumor volume reached approximately 50 mm^3^, the mice were separated into three groups (*n* = 5): (a) control group (5% DMSO), (b) HCPT (3 mg/kg) + SeSAHA (20 mg/kg) group, and (c) SeSA-HCPT (20 mg/kg) group. SeSAHA and SeSA-HCPT were administered by oral gavage, while HCPT was given via tail vein injection. Tumor dimensions were measured every five days in two perpendicular directions, and volumes were calculated using the following formula: volume = (length × (width)^2^)/2. The mice were euthanized on day 22 after drug treatment.

#### Immunohistochemistry

Paraffin-embedded tumor sections were deparaffinized, rehydrated, and subjected to antigen retrieval. After blocking, the sections were incubated overnight at 4 °C with primary antibodies against Ki67 and γ-H2AX. Following PBST washes, the sections were processed using an anti-mouse/rabbit universal immunohistochemical detection kit and counterstained with hematoxylin.

#### Drug toxicity evaluation

Blood samples were collected from each mouse 24 h after the last drug administration and allowed to stand at room temperature for 3 h. The samples were then centrifuged at 3000 rpm for 15 min to obtain serum. The collected serum was subsequently subjected to biochemical analysis using an automated blood analyzer to assess routine blood parameters and evaluate potential drug-induced toxicity.

### Quantification and statistical analysis

All experiments were performed in triplicate. Data are presented as the mean ± SD. Statistical analysis was performed using GraphPad Prism 7.0, including one-way ANOVA and Student’s *t* test. A *p*-value of less than 0.05 was considered statistically significant, with significance levels denoted as ∗*p* < 0.05, ∗∗*p* < 0.01, and ∗∗∗*p* < 0.001.
