# Supplementary material for: SeSA-HCPT: A dual-targeting agent that induces DNA damage and inhibits repair for castration-resistant prostate cancer therapy
Source: iScience. 2026 Jan 29;29(2):114824. doi: 10.1016/j.isci.2026.114824 (PMC12907887; doi:10.1016/j.isci.2026.114824)
Supplement: Document S1. Figures S1–S4 and Table S1 [file mmc1.pdf]

iScience, Volume *29*

Supplemental information

SeSA-HCPT: A dual-targeting agent that induces DNA damage and inhibits repair for castration-resistant prostate cancer therapy

Yajie Wang, Qiuyu Wang, Li Meng, Xiaoying Lian, Xinyue Wu, Yuqing Wang, Tianyu Zhang, ShiLin Wei, Yanming Wang, and Changjun Zhu

Table S1. Antiproliferative Activities of Compounds DmSeSAHA, HCPT, Combined Treatments, and SESA-HCPT Against Various Cancer Cell Lines

Cell lines	***IC_{50} (μM)***			
	DmSeSAHA	***HCPT***	***Combined***	***SESA-HCPT***
Bel-7402	>20	>30	>10	>10
HCT116	1.53±0.108	9.7±0.352	4.2±0.242	3.09±0.152
A549	1.39±0.211	5.12±0.21	0.96±0.12	0.78±0.063
MCF7	1.53±0.183	3.09±0.142	0.89±0.261	0.46±0.082
MDA-MB-231	1.18±0.072	2.12±0.103	0.53±0.075	0.49±0.16
U2OS	2.56±0.136	4.63±0.132	0.42±0.032	3.2±0.287
HeLa	0.61±0.095	2.31±0.086	0.69±0.021	0.131±0.012
PC3	2.73±0.048	12.46±0.752	***1.79±0.094***	***0.198±0.068***
DU145	1.93±0.276	8.52±0.241	***1.754±0.102***	***0.175±0.043***
LNCaP	2.43±0.19	9.63±1.51	***3.768±0.185***	***0.182±0.093***
HaCaT	8.56±0.405	40.13±0.68	***3.04±0.175***	***5.17±0.82***

Note: The data are presented as mean ± SD derived from dose-response curves obtained from at least three independent experiments.

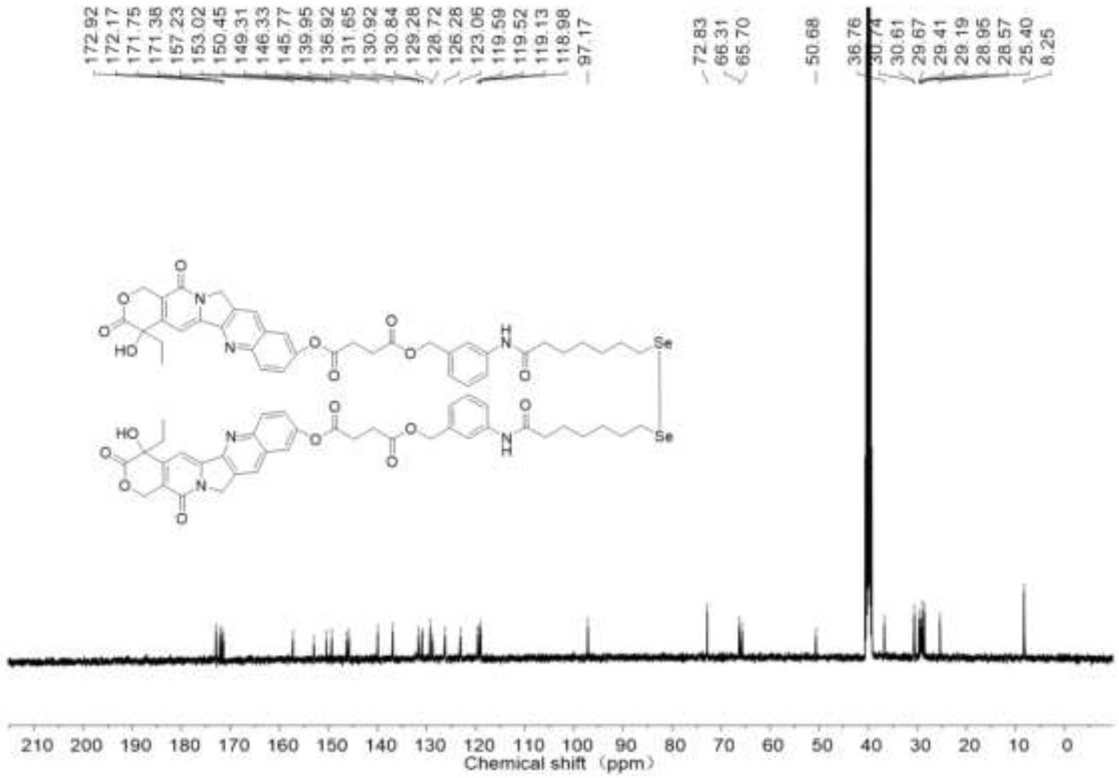

Figure S1. **^{14}C NMR spectrum of SeSA-HCPT in *DMSO-d6***

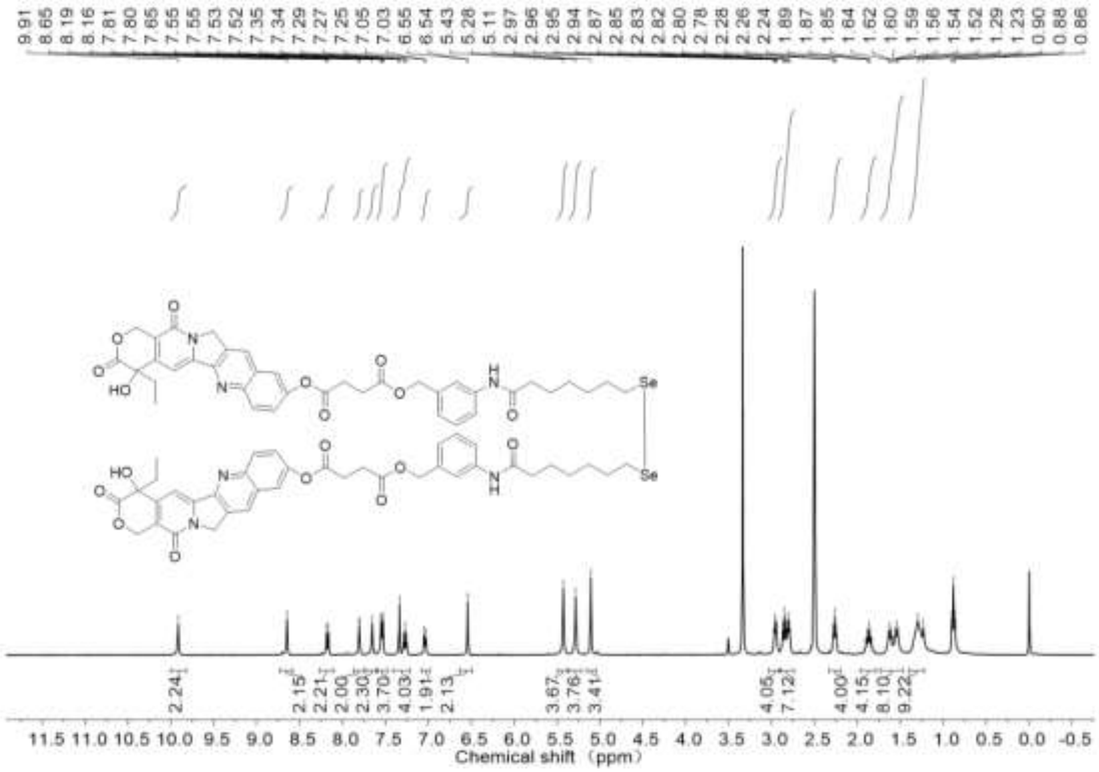

Figure S2. ^{1}H NMR spectrum of SeSA-HCPT in *DMSO-d6*

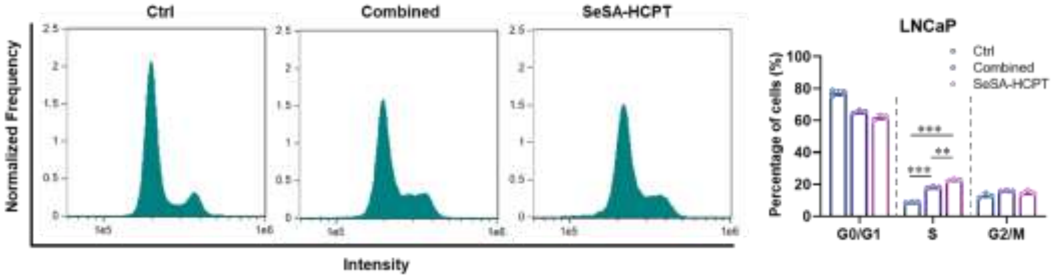

Figure S3. SeSA-HCPT alters cell cycle distribution in LNCaP cells.

LNCaP cells were treated with 200 nM SeSA-HCPT or a combination of agents (200 nM SeSAHA + 400 nM HCPT), with DMSO used as the control. Cell cycle distribution of LNCaP cells was analyzed by flow cytometry. Bar graphs show the percentages of cells in different cell cycle phases under each treatment condition. Data are presented as mean ± SD. Statistical significance is indicated as follows: *P < 0.05, **P < 0.01, ***P < 0.001.

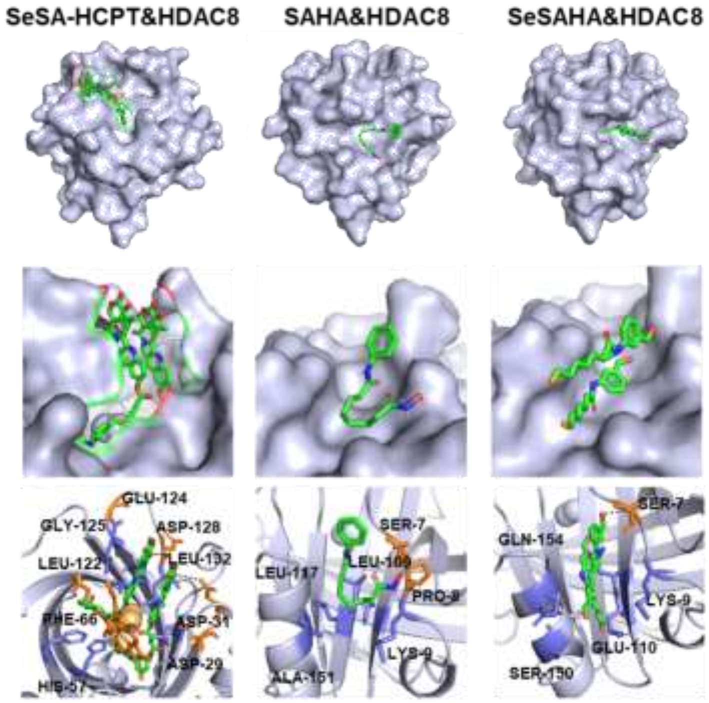

Figure S4. Molecular docking of HDAC8 with compounds.

Transparent surface view images and active site binding diagrams of HDAC8 complexed with SeSA-HCPT, SAHA, or SeSAHA. The panels display both the overall active site pocket topology and the key binding interactions, with orange indicating the positions of hydrogen bond formation.
